# An Abnormal Nitric Oxide Metabolism Contributes to Brain Oxidative Stress in the Mouse Model for the Fragile X Syndrome, a Possible Role in Intellectual Disability

**DOI:** 10.1155/2016/8548910

**Published:** 2015-12-14

**Authors:** Elena Lima-Cabello, Francisco Garcia-Guirado, Rocio Calvo-Medina, Rajaa el Bekay, Lucia Perez-Costillas, Carolina Quintero-Navarro, Lourdes Sanchez-Salido, Yolanda de Diego-Otero

**Affiliations:** ^1^UGC Salud Mental, Hospital Regional Universitario de Malaga, IBIMA Institute, University of Malaga, Research Laboratory, Hospital Civil, Pabellon 5, Sotano, 29009 Malaga, Spain; ^2^Plant Reproductive Biology Group, Department of Biochemistry, Cell and Molecular Biology of Plants, Estacion Experimental del Zaidin, Spanish Council for Scientific Research (CSIC), Profesor Albareda, 1, 18160 Granada, Spain; ^3^UGC Pediatria, Seccion Neuropediatria, Hospital Regional Universitario Malaga, Hospital Materno-Infantil, Planta Baja, 29009 Malaga, Spain; ^4^UGC Endocrinologia y Nutricion, Instituto de Investigacion Biomedica de Malaga (IBIMA), Hospital Regional Universitario de Malaga, Universidad de Malaga, CIBER de Fisiopatología de la Obesidad y Nutricion (CIBERobn), Instituto de Salud Carlos III (ISCIII), Hospital Civil, Pabellon 5 Sotano, 29009 Malaga, Spain; ^5^Departamento de Salud Publica y Psiquiatria, Universidad de Malaga, Teatinos, 29010 Malaga, Spain

## Abstract

*Background.* Fragile X syndrome is the most common genetic cause of mental disability. Although many research has been performed, the mechanism underlying the pathogenesis is unclear and needs further investigation. Oxidative stress played major roles in the syndrome. The aim was to investigate the nitric oxide metabolism, protein nitration level, the expression of NOS isoforms, and furthermore the activation of the nuclear factor NF-*κ*B-p65 subunit in different brain areas on the fragile X mouse model.* Methods.* This study involved adult male Fmr1-knockout and wild-type mice as controls. We detected nitric oxide metabolism and the activation of the nuclear factor NF-*κ*Bp65 subunit, comparing the mRNA expression and protein content of the three NOS isoforms in different brain areas.* Results.* Fmr1-KO mice showed an abnormal nitric oxide metabolism and increased levels of protein tyrosine nitrosylation. Besides that, nuclear factor NF-*κ*B-p65 and inducible nitric oxide synthase appeared significantly increased in the Fmr1-knockout mice. mRNA and protein levels of the neuronal nitric oxide synthase appeared significantly decreased in the knockout mice. However, the epithelial nitric oxide synthase isoform displayed no significant changes.* Conclusions.* These data suggest the potential involvement of an abnormal nitric oxide metabolism in the pathogenesis of the fragile X syndrome.

## 1. Introduction

Fragile X syndrome (FXS) is a rare genetic disorder, mostly characterized by moderate to severe mental retardation, autistic and hyperactive behaviour, macroorchidism, large ears, a prominent jaw, and high-pitched jocular speech [1]. Neuropathological features of the fragile X syndrome are long, thin, and sinuous dendritic spines, increased intracranial volume, enlarged ventricles, increased volumes of selective subcortical grey matter regions, decreased size of the posterior cerebellar vermis, tonic-clonic seizures, and an altered brain glucose metabolism [2, 3]. It is caused by the lack of expression of the fragile X mental retardation protein (FMRP), an mRNA-binding protein encoded by the fragile X mental retardation 1 (FMR1) gene, which is believed to play a role in the regulation of local protein synthesis and possibly mRNA trafficking in the brain [4].

Nitric oxide (NO) is an important signalling molecule that is widely used in the nervous system. NO is synthesized by three different NO synthase (NOS) isoenzymes, all of which are present in the central nervous system (CNS). It is suggested that nitric oxide plays an important role regulating cellular adaptations, and controlling a range of processes in the body, including intracellular signalling, immune function, tissue turnover, expression of antioxidant enzymes, and cellular inflammation. Its involvement in learning, memory, behavioural processes, and cognition is clearly described [5].

With recognition of its roles in synaptic plasticity (long-term potentiation; long-term depression) and elucidation of calcium-dependent NMDAR-mediated activation of neuronal nitric oxide synthase (nNOS), numerous molecular and pharmacological tools have been used to explore the physiology and pathological consequences for nitrergic signalling. In addition, the inability to constrain NO diffusion suggests that spillover from endothelium (eNOS) and/or immune compartments (iNOS) into the nervous system provides potential pathological sources of NO, where control failure in these other systems could have broader neurological implications [6, 7]. However, high levels of NO production also lead, by reaction with reactive oxygen species (ROS), to the formation of peroxynitrite, a highly reactive species contributing to brain oxidative damage and protein nitration. Abnormal NO signalling could therefore contribute to a variety of neurodegenerative pathologies such as intellectual disabilities, stroke/excitotoxicity, Alzheimer's disease, multiple sclerosis, and Parkinson's disease. iNOS is primarily induced by ROS and cytokines through activation of the nuclear factor *κ*B (NF-*κ*B), a ubiquitous transcription factor that plays a key role in regulating immune and inflammatory responses. Under normal conditions, NF-*κ*B is present in the cytoplasm as an inactive heterotrimer [8].

Moreover, oxidative stress leads to increases in ROS, which activates an I*κ*B kinase complex (IKKs), triggering its degradation and allowing free NF-*κ*B to translocate to the nucleus and activate gene expression [9, 10]. NF-kappa-B is a ubiquitous pleiotropic transcription factor that regulates many biological processes such as inflammation, immunity, differentiation, cell growth, tumorigenesis, and apoptosis. NF-kappa-B is a homo- or heterodimeric complex and the heterodimeric p65-p50 complex is the most abundant in the cell. The dimers bind at kappa-B sites in the DNA of their target genes and the individual dimers can bind with different affinity and specificity [11]. Concerning the brain, NF-*κ*B has been implicated in normal processes of synaptic plasticity and memory but also in molecular mechanisms involved in neurological diseases [12].

In this study we analysed whether FXS could be caused by an alteration of NO metabolism, by studying the activation of the NF-*κ*B-p65 subunit, the most abundant activated form in the nucleus, and also 3-nitrotyrosine and NO level, together with NOS isoform proteins detection in different brain areas from Fmr1-KO compared with wild-types of different ages. This work presents NO metabolism abnormalities in the Fmr1-KO mouse brain; these findings seem to suggest that NO may be involved in the pathogenesis of FXS and may represent a new therapeutic pathway for research.

## 2. Material and Methods

### 2.1. Experimental Animal Model

Animal studies were approved by the Animal Care and Use Committee of the Carlos Haya Hospital in accordance with the National Institute of Health guidelines for animal care. The Fmr1-knockout FVB-129 mouse line, donated by B. Oostra (Erasmus University Rotterdam), was used for the experiments. The animals were housed as a mouse colony in the Experimental Animal House of the University of Malaga. All experimental protocols met the guidelines of the University of Malaga Animal Welfare Committee and the European Communities Council Directive (86/609/EEC) regarding the handling of experimental animals. The mice were housed under controlled conditions of temperature and humidity, with a 12 h light/dark cycle with free access to standard food and water. Wild-type (WT) and Fmr1-knockout (Fmr1-KO) mice at 3 and 6 months of age were used in these experiments. Mouse littermates were obtained from crossings of heterozygous (wild-type/Fmr1-knockout) females and hemizygous (Fmr1-knockout) males and randomly located in different experimental groups, with 8–12 animals per group. The animals were sacrificed by cervical dislocation. Mouse brains were dissected immediately onto a cold plate, frozen, and stored at −80°C for biochemical analysis.

### 2.2. Genotype Analysis

DNA from the tail tissue was isolated by the Salting Out protocol to analyse genotype. The knockout mutation was distinguished from the normal gene by PCR analysis following the procedure used in previous publications [13]. Diluted DNA (2 *μ*L) and 5 pmol of appropriate primers were used with the Ready Mix Kit (Sigma, S. Louise, MO). The PCR reactions were carried out on a thermocycler (Perkin-Elmer, Fremont, CA) and the products were visualized using ethidium bromide in a 1.5% agarose gel electrophoresis.

### 2.3. Brain Dissection

Animals were sacrificed by cervical dislocation. The brains were removed immediately, frozen promptly in liquid nitrogen, and the different regions, hippocampus, prefrontal cortex, and cerebellum, were dissected following Paxino's atlas coordinates and stored at −80°C.

### 2.4. LPS-Activated Organotypic Culture Brain Slice

For* ex vivo* experiments, the brain was cut into 100 *μ*m thick slices and transferred onto plates containing sterile cold KR-HEPES buffer for assays. Organotypic slices culture of the central nervous system was performed following the standardized protocol. Incubations of the organotypic slice culture in the presence of 30 mg/mL or 60 mg/mL* Escherichia coli* lipopolysaccharide (LPS) exotoxin were carried out to induce inducible nitric oxide synthase (iNOS) expression. Organotypic culture slices were incubated for 1 hour in the presence of 300 mM aminoguanidine (AG) to inhibit the iNOS, and then 30 mg/mL or 60 mg/mL LPS was added. The NO production was measured in the culture medium in untreated or treated organotypic slices culture for 10 min with LPS in the absence or presence of 300 *μ*M AG and the results obtained were expressed as *μ*M of nitrite/nitrate production.

### 2.5. Determination of Nitrate Concentration

Nitrate concentration was measured in the different brain areas by using a commercial assay kit (Abcam, Cambridge, UK).

### 2.6. Relative Quantification of nNOS, iNOS, and eNOS mRNAs

Real-time quantitative PCR technology was used to assay nNOS, iNOS, and eNOS mRNAs expression on 10 samples from each experimental group. Total RNA was isolated from the different brain areas by using the RNeasy Lipid Tissue RNA isolation kit (Quiagen, Netherlands). First strand cDNA was synthesized using High-Capacity cDNA Archive Kit (Applied Biosystems, Weiterstadt, Germany). For gene expression assays, cDNA was amplified using the 7500 Fast Real-Time PCR System (Applied Biosystems). Primers and probes were used from the commercially available TaqMan Gene Expression Assays (Mm 00607939_s1, Mm00435217_m1, Mm00440502_m1, and Mm00435175_m1, Applied Biosystems). (Relative changes in gene expression levels were determined using the 2^−ΔΔCt^ method). The cycle number at which the transcripts were detectable (CT) was normalized to the cycle number of GAPDH detection, referred to as ΔCT. PCR efficiency was determined by TaqMan analysis on a standard curve for targets and endogenous control amplifications, which were highly similar.

### 2.7. Western Blot Analysis

Western blot analyses were performed on brain extracts. Mouse brains were dissected into main structures comprising the cortex, hippocampus, and cerebellum. Samples were homogenized using a motorized pellet pestle in 1 mL of homogenization buffer containing 20 mM HEPES and 100 mM KCl pH 7.0 with protease inhibitor cocktails (SigmaFast, cat# 8830, Sigma, Saint Louis, MO). Cytosolic fractions were isolated by centrifugation. Protein concentration in each sample was measured by the Bradford method. Aliquots of tissue samples corresponding to 40 *μ*g (cytosol) and 20 *μ*g (nucleus) were heated to 100°C for 5 min with an equivalent volume of sample buffer (containing 2% SDS and 5% mercaptoethanol, bromophenol blue, and 20% glycerol) and loaded onto 10% and 12% polyacrylamide gels. Proteins were electrotransferred to a PVDF membrane, blocked for 1 h at 37°C in a blocking solution containing 3% BSA, 0.05% Tween-20, and PBS (pH 7.4), and incubated overnight at 4°C with primary antibodies in the blocking solution. An antibody against 3-nitrotyrosine was purchased from BIOMOL International (Plymouth Meeting, PA); antibodies against nNOS (155 kDa), iNOS (130 kDa), and eNOS (133 kDa) were purchased from Abcam (Cambridge, UK) and an antibody against p65 (65 kDa) was purchased from Santa Cruz Biotechnology (Santa Cruz, CA). Membranes were rinsed three times with 0.05% Tween-20 in PBS for 10 min each, followed by incubation for 1 h at room temperature in a 1 : 5000 dilution of goat anti-rabbit IgG-HRP, purchased from Santa Cruz Biotechnology (Santa Cruz, CA), with 3% BSA in PBS. The blot was washed three times for 10 min each and then processed for analysis using an enhanced chemiluminescence detection kit (Biorad, cat# 170-5070) and visualized with a digital luminescent image analyser (Biorad).

### 2.8. Statistical Analysis

Data are expressed as means ± SEM of 5-6 mice for group. Two-tailed Student's *t*-test was used to compare the two groups. One-way ANOVA followed by Dunnett's test was used to compare among three or more study groups. *P* < 0.05 is regarded as statistically significant.

## 3. Results

### 3.1. Basal Levels of Nitrite/Nitrate Were Reduced in the Cytosolic Fraction of Different Brain Areas

**Figure 1:**
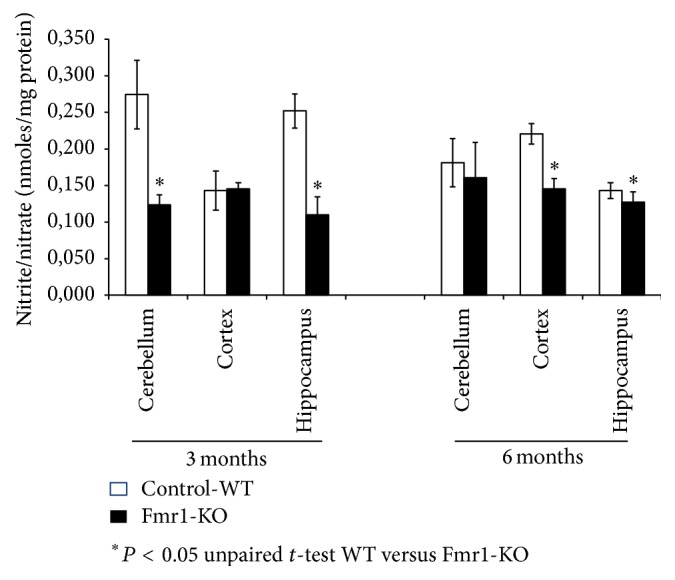
Nitrite/nitrate concentration in the cytosolic fraction of different brain areas, such as hippocampus, cortex, and cerebellum, and from fragile X mental retardation 1-knockout (Fmr1-KO) and wild-type (WT) mice of different ages. Data are described as the mean values ± standard deviation of eight experiments (^*∗*^ *P* < 0.05 versus WT).

Figure 1 shows the NO concentration in different brain areas, such as hippocampus, prefrontal cortex, and cerebellum. A total of 6 mice samples were grouped by age at different stages (3 and 6 months). The results showed that the production of NO in each of the studied areas and at the different ages from Fmr1-KO mice displayed a lower production of NO when compared to WT-controls. NO production was significantly lower in Fmr1-KO mice compared to WT in cerebellum and hippocampus of the 3-month-old mice, and no changes occurred in cerebellum from 3-month-old mice. Moreover, NO production was significantly reduced in cortex and hippocampus of 6-month-old Fmr1-KO mice compared to WT.

### 3.2. Nitric Oxide Metabolism Was Increased in LPS-Activated Organotypic Culture Brain Slices Obtained from the Fmr1-KO Mouse

Inducible nitric oxide metabolism was studied in the central nervous system using organotypic culture brain slices, under different incubation conditions to analyse if the NOS2 enzyme was involved in NO production in the Fmr1-KO mouse brain.

**Figure 2:**
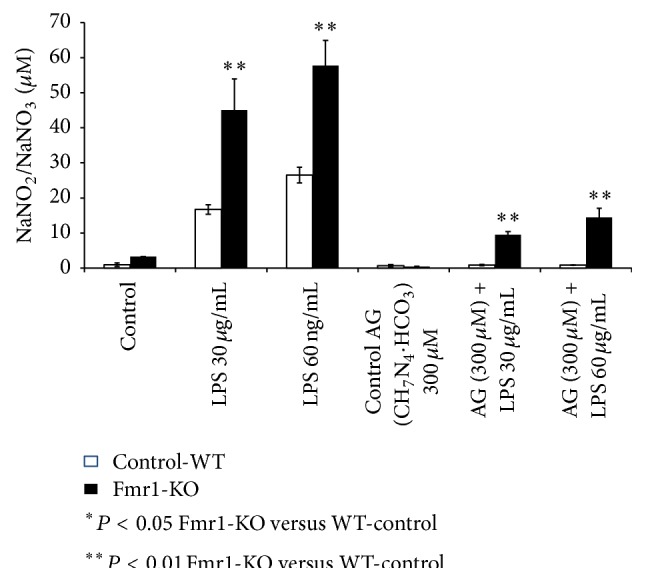
NO production is enhanced after lipopolysaccharide incubation and inhibited by aminoguanidine (AG) treatment of organotypic cultured brain slices from the Fmr1-KO mice. NO analysis was performed in culture brain slices by a nitrate/nitrite colorimetric test kit in the culture medium. Data represent mean ± SEM. ^*∗*^ *P* < 0.05 FXS versus control. ^*∗∗*^ *P* < 0.01 FXS versus control.

The incubation of culture slices in the presence of LPS increased the production of NO in a concentration dependent manner in the Fmr1-KO mouse, as can be observed in Figure 2.

The inhibition of the NO production by a NOS2 inhibitor, such as AG, confirms the extremely significant reduction of NO production in culture slices incubated for one hour in AG before the incubation in the presence of LPS. However, the complete inhibition was not seen within our experimental conditions.

### 3.3. 3-Nitrotyrosine Is Enhanced in Brain Proteins

**Figure 3:**
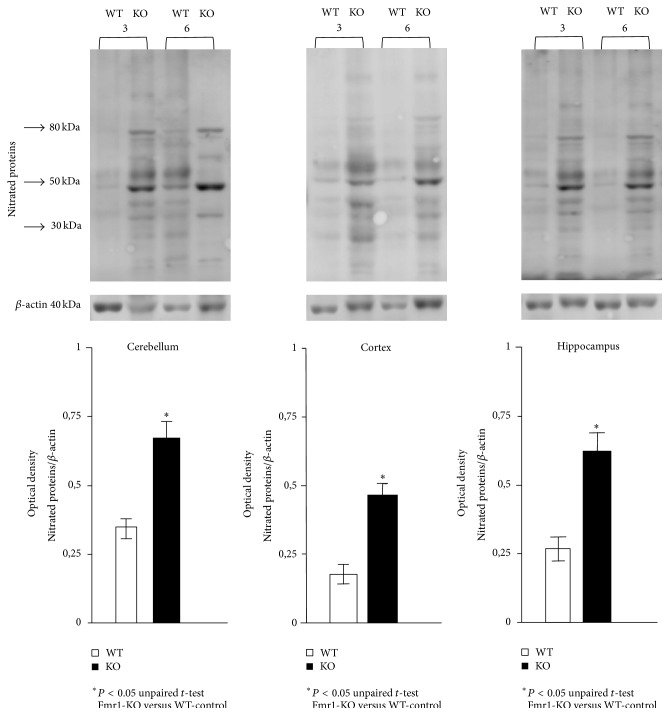
Western blot analysis of nitrated proteins in the cytosolic fraction of different brain areas, such as hippocampus, cortex, and cerebellum from fragile X mental retardation 1-knockout and wild-type (WT) mice. Molecular weight markers (kDa) are indicated on the left. Photographs are representative of six independent experiments. Data are described as the mean values ± standard deviation of six experiments (^*∗*^ *P* < 0.05 versus WT).

NO is an important signalling molecule that is widely used for signalling during normal physiological processes of the nervous system. Superoxide and NO are known to bind to form peroxynitrite. Peroxynitrite and/or peroxynitrite-derived intermediates can free nitrate or protein-bound tyrosine residue to form 3-nitrotyrosine. The detection of protein-bound 3-nitrotyrosine is often used as a marker of inflammation and NO overproduction.  Figure 3 shows the pattern of nitrated proteins in different brain areas, such as hippocampus, prefrontal cortex, and cerebellum. Detected immunoreactive bands indicate a statistically significant increase of endogenous tyrosine nitration in each studied area from the Fmr1-KO mice when compared to WT-controls at the two different adult stages.

### 3.4. NF-*κ*B-p65 Subunit Is Upregulated in the Brain

**Figure 4:**
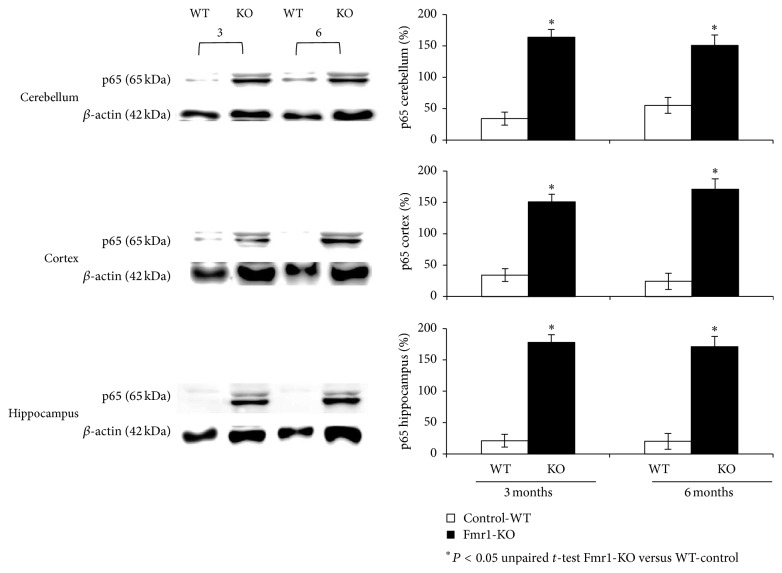
Western blot analysis of p65 protein levels in the nuclear fraction of different brain areas, such as hippocampus, cortex, and cerebellum from fragile X mental retardation 1-knockout (Fmr1-ko) and wild-type (WT) mice of different ages. Densitometry analysis of specific bands expressed as percentage relative to WT samples (100%). *β*-actin levels were used as loading control. Molecular weight markers (kDa) are indicated on the left. Photographs are representative of six independent experiments. Data are described as the mean values ± standard deviation of six experiments (^*∗*^ *P* < 0.05 versus WT).

Most of the NF-*κ*B target genes encode proteins participating in stress and inflammatory responses, such as iNOS which is known to have NF-*κ*B binding sites on its promoter. Thus NF-*κ*B seems to be a key molecular mechanism for iNOS mRNA induction, and its activation seems to be a beneficial mediator to cellular stress. To determine the possible activation of the NF-*κ*B-p65 subunit, Western blot analyses were performed in cortex, hippocampus, and cerebellum tissues.  Figure 4 shows that, in the three studied regions, NF-*κ*B-p65 protein content was significantly higher in Fmr1-KO mice when compared to WT mice.

### 3.5. nNOS and iNOS Protein Levels Are Divergent in the Brain

**Figure 5:**
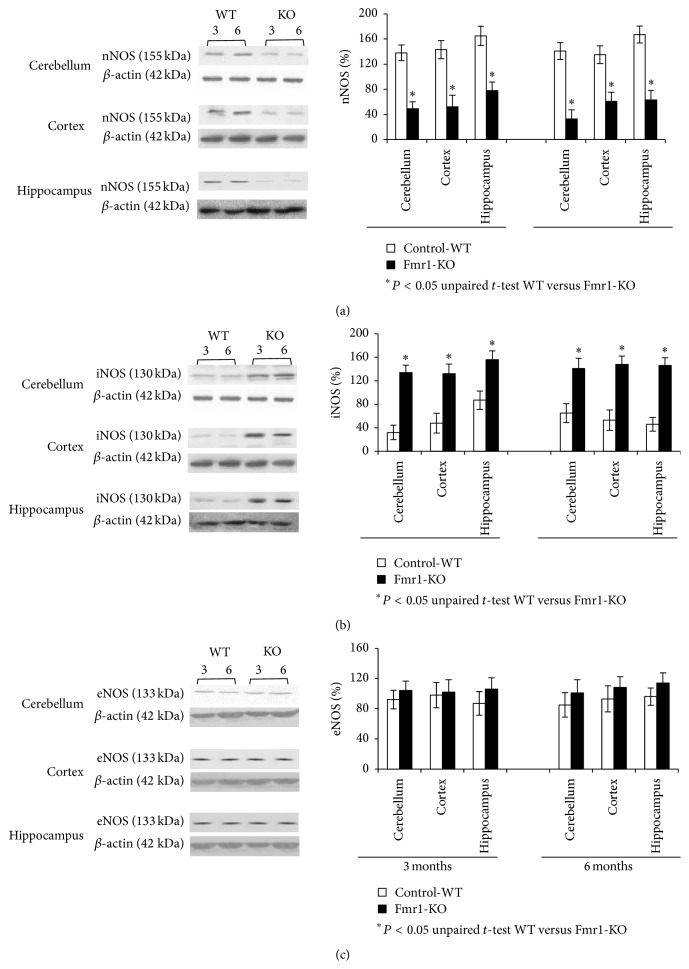
((a), (b), and (c)) nNOS, iNOS, and eNOS protein levels in the cytosolic fraction of different brain areas, such as hippocampus, cortex, and cerebellum from fragile X mental retardation 1-knockout (Fmr1-KO) and wild-type (WT) mice of different ages, were analysed by Western blot. Densitometry analysis of specific bands expressed as percentage relative to WT samples (100%). *β*-actin levels were used as loading control. Molecular weight markers (kDa) are indicated on the left. Photographs are representative of six independent experiments. Data are described as the mean values ± standard deviation of six experiments (^*∗*^ *P* < 0.05 versus WT).

In order to further investigate the role of NO in the fragile X mouse pathology, we analysed whether NOS isoforms are activated in the brain from fragile X syndrome mice. Here we studied the activation of three different proteins: nNOS, iNOS, and eNOS by using the immunoblotting technique and also real-time PCR to measure mRNA expression levels. We first analysed NOS isoforms activation in different brain areas, such as hippocampus, prefrontal cortex, and cerebellum, and in different ages from Fmr1-KO animals and WT. Fmr1-KO mice displayed a significant decrease in nNOS protein content in all the different areas of 3- and 6-month-old Fmr1-KO mice when compared to WT-controls. However, this decrease was more evident in the cortex at both ages than in the hippocampus and in the cerebellum (Figure 5(a)). The iNOS isoform was highly expressed in the three studied areas from Fmr1-KO mice at both ages when compared to same aged WT-controls (Figure 5(b)). Regarding eNOS protein content from 3- and 6-month-olds, no significant differences were found between the three studied areas of Fmr1-KO mice compared to WT-control group (Figure 5(c)).

### 3.6. Abnormal Expression Pattern of NOS Isoforms in the Brain

**Figure 6:**
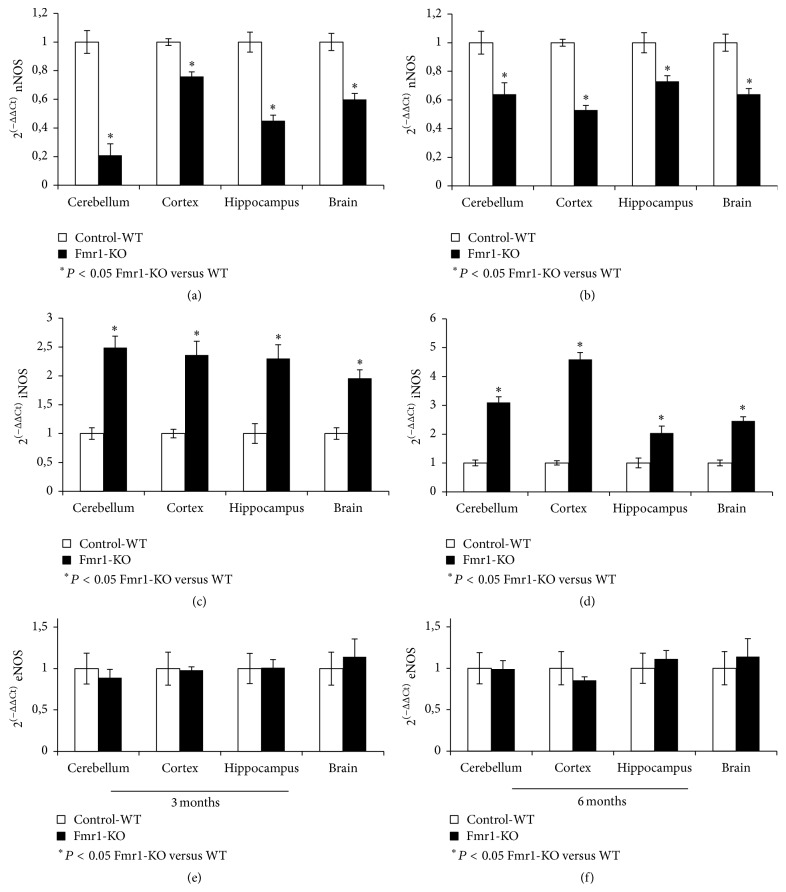
mRNA levels determined by real-time qRT-PCR of the three NOS isoforms in (a), (b), (c), (d), (e), and (f). Bar graph shows nNOS, iNOS, and eNOS in the different brain areas, such as hippocampus, cortex, and cerebellum from fragile X mental retardation 1-knockout (Fmr1-KO) and wild-type (WT) mice of different ages (^*∗*^ *P* < 0.05 versus WT).

To further characterize the involvement of these proteins in the fragile X phenotype, we analysed the mRNA expression level of the three proteins in the same studied areas and in whole brain.  Figure 6 shows real-time PCR experiments of the three proteins normalized by GADPH mRNA levels. Figures 6(a) and 6(b) demonstrate a significant decrease in the levels of mRNA nNOS expression in the whole brain and in the three selected areas, when comparing Fmr1-KO samples at both age groups compared with WT-control samples. The decrease was particularly pronounced in cerebellum of 3-month-old Fmr1-KO compared to WT (Figure 6(a)). The mRNA level of the iNOS was significantly increased in whole brain and in the three selected areas, when comparing 3- and 6-month-old Fmr1-KO with WT-controls (Figures 6(c) and 6(d)). Surprisingly, this effect was more evident in the hippocampus of 6-month-old Fmr1-KO compared to WT than in the rest of the studied areas of this same group (Figure 6(d)). Regarding gene expression of the eNOS isoform, no changes were observed in whole brain and in the three different selected areas of 3- and 6-month-old mice of the Fmr1-KO and WT group (Figures 6(e) and 6(f)).

## 4. Discussion

**Figure 7:**
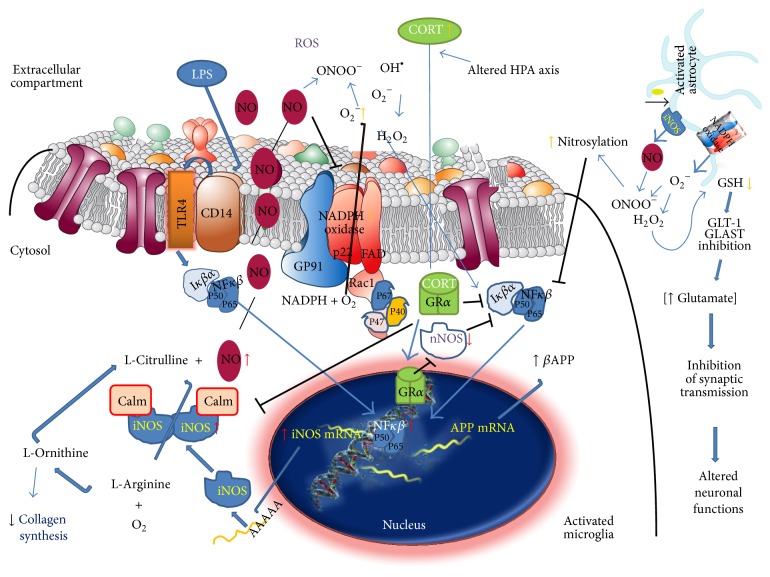
Glucocorticoids (Cortisol/Corticosterone) act as a nNOS inhibitor, and both molecules induce the releases of the NF-*κ*B transcription factor that translocate to the nucleus to activate the synthesis of iNOS. Also other extracellular signals, such as lipopolysaccharide, act to induce NO production. NO acts as neurotransmitter and neuromodulator that regulates the NMDA receptors in the nervous systems. nNOS production of NO in CNS is associated with pain perception, control of sleep, appetite, thermoregulation, neural development, and synaptic plasticity. Activated microglia secrete inflammatory mediators to coactivate astrocytes and to induce cellular damage, which in turn will lead to abnormal synaptic transmission and neuronal dysfunction. NO antagonises NADPH oxidase (NOX2) assembly that in turn leads to a reduction in ROS production. Thus, as NO levels decline, oxidative mechanisms increase. Oxidative and nitrosative stress can also decrease intracellular GSH (reduced form) levels, resulting in a reduced antioxidant capability of the cells and an abnormal neuronal function. NO enhances glutamate release to influence memory processes by long-term potentiation (LTP). NO has been suggested as the retrograde molecule that activates the glutamate release. A compensatory mechanism, involving nNOS, is present in order to maintain LTP. The effect of NO on glutamate release depends on the NO level. Thus, when NO concentrations are low there is a decrease in glutamate release, but when NO increases, the inhibitory effect on glutamate release is reversed. S-Nitrosylation is a posttranslational regulatory mechanism that reduces the activity of target proteins, such as ERK, GAPDH, caspases, transglutaminases, and GTPase proteins (p21, RAC1, or cdc42). It has also been demonstrated that NO can induce S-nitrosylation and downregulate the NMDA receptor. S-Nitrosylation inhibits NF-*κ*B, which could be an autoregulatory mechanism of NO production, since iNOS is induced by this transcription factor. NMDA receptor activation leads to the activation of nNOS, which could play a protective role by S-nitrosylation of the NMDA receptor; this inhibitory mechanism is regulated in order to prevent toxic effects. However iNOS activation and NO overproduction have also been suggested as an activator of NMDA-dependent neurotoxicity. Also, NO synthesis is involved in collagen physiology leading to connective tissue abnormalities.

ROS are emerging as important players in the aetiology of neurologic and neurodevelopmental disorders including FXS. Out of several ROS-generating systems, the enzymes nicotinamide adenine dinucleotide phosphate (NADPH) oxidase and NOS are believed to play major roles; and Figure 7 illustrates a diagram including key components of the molecular signalling mediated by this pathway. Mounting evidence suggests that activation of NADPH oxidase and the expression of NOS are directly linked to the generation of highly reactive free radicals, which affect various cellular components. Our previous studies carried out on the Fmr1-KO indicated that brain oxidative stress (increased generation of ROS, activation of the enzyme NADPH oxidase) is involved in several pathological manifestations of the syndrome [14–16]. In the present study, we demonstrate that adult Fmr1-KO mice develop altered NO homeostasis. In addition NO is a physiological messenger molecule; NO has been shown to participate in many physiological processes as signalling molecule and in long term it is involved in several pathophysiological mechanisms in the brain. Various forms of neuronal injury are frequently associated with increased or de novo overexpression of NOS [17]. On the basis of animal experiments, NO has been implicated in molecular mechanisms of epileptic seizures, ranging from mediation of an excitotoxic cascade to modulation of the central nervous system blood flow during epileptic episodes, and finally to participation in subsequent neuronal injury [18]. During pathologically increased overproduction of NO, much of the newly synthesized NO will be converted into peroxynitrite, which is an extremely potent free radical [19]. This substance subsequently interferes with mitochondrial energy metabolism and may even cause death of neurons by necrosis or apoptosis [20]. In this scenario, we have described an overproduction of NO after lipopolysaccharide incubation and the increased NO level was inhibited by aminoguanidine treatment of organotypic cultured brain slices from Fmr1-KO mice. Further studies must be carried out to understand if the inhibition of nNOS, by an excessive NADPH oxidase free radical overproduction, could constitute a cytoprotective mechanism against the negative consequences of the continual enhancement of oxidative molecules, which provides a novel clue to extend the physiological and pathophysiological roles of oxidative stress in the fragile X syndrome.

In our study, the level of 3-nitrotyrosine, another marker related to nitroxidative stress, was significantly elevated in cortex cerebellum and hippocampus homogenates of Fmr1-KO mice when compared to WT-controls. Most brain pathologies are accompanied by inflammation, during which the production of NO (mainly from iNOS) and/or superoxide (O_2_^−^) plus H_2_O_2_ (mainly from NOX) is increased [21].

iNOS is not normally expressed in the brain, but inflammatory mediators such as lipopolysaccharide and cytokines cause its expression in microglia and astrocytes, and possibly in neurons [22]. Indeed, we found a statistically significant increase in inducible iNOS isoform levels in Fmr1-KO mice compared to WT in young and adult mice in the three different studied areas.

Our results indicate an overall increased level of NF-*κ*B-p65 in the brain from Fmr1-KO mice. Our main goal attempts to suggest a cooperative role of NO and NF-*κ*B by regulating or contributing to the oxidative stress described in the brain of the Fmr1-KO mouse. Nuclear transcription factor kappa B is a stress inducible transcription factor, and it was demonstrated previously by others [23] that mice lacking a subunit of NF-*κ*B-p65 show a selective learning deficit in the spatial version of the radial arm maze. These observations suggest that long-term changes of this factor affect adult neuronal function after synaptic stimulation, and it can be regulated by NF-*κ*B nuclear translocation leading to gene activation, such as iNOS. It has been suggested that NF-*κ*B activity following hippocampal learning may contribute to consolidation-associated synaptic reorganisation [24]. Moreover, the possible role of NO and NF-*κ*B in the locomotor activity of rats has been described [25], and Fmr1-KO mice showed significant hyperactivity during development is frequently found in fragile X patients [13]. Kaltschmidt and cols. [26] have speculated that changes during cerebellar development may be controlled by glutamate-induced gene expression involving NF-*κ*B. Similarly, the important role of NO in cell development was described in several animal models and humans [27].

The pathological effects of abnormal expression of NF-*κ*B may result in many diseases. Those molecular mechanisms have not yet been thoroughly studied in the fragile X syndrome. We provide data to support the role of NF-*κ*B in the Fmr1-KO mouse model. To the best of our knowledge, no study has yet been reported on NF-*κ*B in children or in the Fmr1-KO mouse model. In our study, we have found that there was a significant increase in the amount of the NF-*κ*B-p65 subunit in nuclear extracts, from cortex, cerebellum, and hippocampus in both ages from Fmr1-KO mice when compared to WT. Elevated amounts of p65 in the Fmr1-KO mouse model can strengthen the conceptual frameworks of the role of free radicals (ROS and RONS) in the etiopathology of this condition. Naik and cols. [28] noted a significant increase in NF-*κ*B DNA binding activity in peripheral blood samples of children with autism. NF-*κ*B regulates a vast number of genes including those encoding cytokines, death and survival proteins, adhesion molecules, cyclooxygenase-2, manganese superoxide dismutase, and, of course, inducible nitric oxide synthase [29]. Both increased expressions of iNOS and NF-*κ*B have been shown to participate in neurodegenerative diseases such as Parkinson's disease, Huntington's disease, or Alzheimer's. Inhibition of NF-*κ*B decreased inducible nitric oxide synthase and cyclooxygenase-2 expression and restored working memory in the Alzheimer's disease mouse model [30].

nNOS is predominantly active in central and peripheral neurons, where production of NO is very important for cell communication [31]. nNOS produces low concentrations of NO over long periods and is activated by calcium ions (Ca^2+^) through transient binding to the calcium-binding protein calmodulin. The results presented here demonstrate that protein and mRNA levels of nNOS of 3- and 6-month-old Fmr1-KO mice were relatively low in the three studied areas compared to WT mice. However, we suggest that the attenuated nNOS expression probably results in downregulation of NO production in Fmr1-KO mice. However, in our study, the production of NO was significantly lower in Fmr1-KO than in WT, in both ages, and in the three studied areas.

This deficiency probably results in an inadequate production of NO during nervous system development. nNOS regulation differs from iNOS; it is tightly regulated by calcium-activated calmodulin, specific phosphorylation, or interaction with plasma membrane ionotropic receptors [32]. This tight regulation makes NO generation an ideal signalling molecule in neurons that can regulate physiological processes such as differentiation and plasticity in the nervous system [33, 34]. L-Arginine is the only endogenous substrate of NOS and thus governs the production of NO during nervous system development. The suppression of nNOS gene expression has been attributed to large amounts of cytokines released during inflammation [35]. It has also been noted that the amount of NO produced by iNOS can depend greatly on the amount of the substrate, l-arginine (l-Arg) [36]. In addition, it has been suggested that NO derived from nNOS activity keeps iNOS suppressed under normal conditions, whereas downregulation of nNOS is a necessary condition to facilitate the expression of iNOS and the release of large amounts of NO [35]. To our knowledge, this is the first study demonstrating dysregulation of iNOS expression in the Fmr1-KO mouse model.

Recently published research has demonstrated that nNOS-knockout mice exhibit higher locomotor activity than their wild-type counterparts in a novel environment, as measured in the open field (OF) test, and also significantly shorter step-through latencies after training in a passive avoidance paradigm. Furthermore, abnormal spontaneous motor activity rhythms were found in the KO during the dark phase of the day, indicating dysregulation of rhythmic activities. These data indicate that nNOS-KO mimics certain ADHD-like behaviours [37]. It has been extensively demonstrated that Fmr1-knockout mice display hyperactivity [13], attentional and learning deficits consistent with a possible implication of a reduction in nNOS expression, and reduced nitric oxide basal production as has been shown in this work.

eNOS produces NO mainly in endothelial tissue of blood vessels, where NO causes vasodilation and endothelial relaxation of muscles and soft tissue [38]. However, in our study no differences were found in the eNOS isoform between different ages or between the Fmr1-KO and WT-control group.

The levels of nitroxidative stress were significantly higher in persons with ID such as Down syndrome and West syndrome [20, 39]. Our results are in agreement with previously published research demonstrating that nitroxidative stress is involved in intellectual disabilities. Xu et al. suggested that nNOS was reduced on postnatal day 21 in the hippocampus of Fmr1-knockout and impaired NO production may retard spine maturation in FXS [40]. Kwan et al. found that neocortical nNOS protein levels are severely reduced in developing human FXS cases [41]. Thus, alterations in regulation of nNOS in brain circuits may contribute to cognitive dysfunction in FXS.

Oxidative stress is involved in the fragile X syndrome as it was previously proposed [13, 42, 43]; it was also demonstrated that antioxidants and free radicals scavengers will be useful as experimental therapeutic approach to treat fragile X pathology [14, 44]. This work proposed that abnormal nitric oxide metabolism is involved in oxidative stress, and this mechanism may benefit antioxidant treatment. Also, selective nitric oxide synthase inhibitors may be investigated to determine the therapeutic possibilities in fragile X syndrome.

## 5. Conclusion

NOS abnormal expression observed in the Fmr1-KO mouse brain leads to changes in the NO metabolism, protein nitration, and NF-*κ*B activation that may be involved in the pathogenesis of intellectual disability in the FXS and may represent a new therapeutic target for this rare disorder. In conclusion, the regulation of both iNOS and nNOS may play a fundamental role in the pathophysiological pathways contributing to FXS. Thus, the cross-talk of iNOS, nNOS, and NF-*κ*B is suggested in neurological processes. Several neurological and inflammatory disorders have been linked to NF-*κ*B, therefore restoring the nitric oxide/nitrosative status, and also to REDOX equilibrium on the FXS which addresses a promising approach for further investigation on treatment trials for the disease as has been also suggested in previous recent publications [45, 46].

## Abbreviations

Calm: Calmodulin
CORT: Cortisol/Corticosterone
NF-*κ*B: Nuclear factor *κ*B
iNOS: Inducible nitric oxide synthase
ROS: Reactive oxygen species
NO: Nitric oxide
GLAST: Glutamate/aspartate transporter
GLT-1: Glutamate transporter subtype 1
GSH: Glutathione
ROS: Reactive oxygen species
LPS: Lipopolysaccharide.
